# Understanding feasibility and acceptability of implementation of linking delivery of family planning and infant vaccination care in rural Maharashtra, India: a qualitative study

**DOI:** 10.1186/s12884-023-05830-z

**Published:** 2023-07-15

**Authors:** Sarah Averbach, Edwin Elizabeth Thomas, Gennifer Kully, Melody Nazarbegian, Mohan Ghule, Borsika A. Rabin, Anita Raj, Nandita Bhan

**Affiliations:** 1 Department of Obstetrics, Gynecology and Reproductive Sciences, University of California, 9300 Campus Point Drive #7433, San Diego, La Jolla, CA 92037-7433 92037 USA; 2 Center on Gender Equity and Health, University of California San Diego School of Medicine, San Diego, La Jolla, CA USA; 3 Center of Gender Equity and Health, University of California San Diego, Delhi, India; 4 University of California San Diego School of Medicine, La Jolla, CA USA; 5 Herbert Wertheim School of Public Health and Human Longevity Science, University of California San Diego, La Jolla, CA USA; 6 UC San Diego Altman Clinical and Translational Research Institute Dissemination and Implementation Science Center, University of California San Diego, La Jolla, CA USA; 7 Jindal School of Public Health and Human Development, OP Jindal Global University, Sonipat, Haryana India

**Keywords:** Postpartum, Contraception, Gender-equity, Community-based care, Implementation science

## Abstract

**Background:**

Linking family planning with infant vaccination care has the potential to increase contraceptive use among postpartum women in rural settings. We explored the multilevel factors that can facilitate or impede uptake of contraception at the time of infant vaccination among postpartum women and couples in rural Maharashtra, India.

**Methods:**

We conducted 60 semi-structured interviews with key stakeholders including: postpartum married women (n = 20), husbands (n = 10), and mothers-in-law (n = 10) of postpartum women, frontline healthcare workers (auxiliary nurse midwives (ANMs) and Accredited Social Health Activists (ASHAs), (n = 10), and community leaders (physician medical officers and village panchayat leaders) (n = 10). We sought to assess the feasibility and acceptability of delivering community-based postpartum family planning care in rural India at the time of infant vaccination. The Consolidated Framework for Implementation Research (CFIR) was used to design a structured interview guide and codebook. Data were analyzed via directed content analysis.

**Results:**

Three major themes emerged: (1) Social fertility and gender norms including son preference and male control over contraceptive decision-making influence postpartum contraceptive access and choice. (2) Linking contraceptive care and infant vaccination is perceived as potentially feasible and acceptable to implement by families, health workers, and community leaders. The intervention provides care to women and families in a convenient way where they are in their community. (3) Barriers and facilitators to linked infant postpartum contraception and infant vaccination were identified across the five CFIR domains. Key barriers included limited staff and space (inner setting), and contraceptive method targets for clinics and financial incentives for clinicians who provide specific methods (outer setting). Key facilitators included convenience of timing and location for families (intervention characteristics), the opportunity to engage husbands in decision-making when they attend infant vaccination visits (participant characteristics), and programmatic support from governmental and community leaders (process of implementation).

**Conclusions:**

Linked provision of family planning and infant vaccination care may be feasible and accessible in rural India utilizing strategies identified to reduce barriers and facilitate provision of care. A gender-transformative intervention that addresses gender and social norms has greater potential to impact reproductive autonomy and couples’ contraceptive decision-making.

## Background

Effective postpartum contraception can prevent short inter-pregnancy intervals (fewer than 24 months between births) which are associated with an increased risk of maternal and infant morbidity and mortality worldwide [1–9] including in India [10]. India is the country with the highest number of women with an unmet need for contraception, and postpartum and rural women are those with the greatest need for these services [11]. Uptake of postpartum contraception among women in rural India is low; it’s estimated that only 14–36% postpartum women use contraception within the first year after delivery, and 27% of births do not follow the recommended inter-pregnancy interval [12–14].

Integration of postpartum family planning into infant vaccination services has been recommended as a strategy to improve family planning uptake worldwide by leveraging existing public health infrastructure. However, the effectiveness of this approach has not be consistently demonstrated [15–18]. In India, immunization services are one of the most widely implemented and successful public health programs [19]. India’s Ministry of Health and Family Welfare promotes community-based delivery of infant vaccinations and monthly childhood vaccination services are offered at Village Health and Nutrition Days (VHNDs). Six-week vaccination rates are high, around 85% [20], indicating that these services are routinely used by families of infants, which provides an opportunity to reach postpartum women and couples outside of health clinics where they are in their communities. Prior research demonstrated that screening for interest in family planning services at VHNDs in India was associated with an increase in women later seeking family planning services through the public health system [15]. However, provision of family planning counseling and contraceptive methods at the time of vaccination services has not been studied in India.

We sought to explore women’s, family’s, and provider’s perspectives on whether and how to integrate family planning into infant vaccination services in rural communities in India. We sought to understand community and provider perceptions of the feasibility and acceptability of community-based delivery of family planning, inclusive of the full range of reversible contraceptive methods, at the time of infant vaccination. We aimed to inform an implementation strategy to support the successful adoption, implementation, sustainment, and scale-up of linked care for broad uptake by rural health systems in India. We aimed to engage potential users of the intervention to inform an implementation strategy that is responsive to the needs of their community.

## Methods

We engaged community members as partners to guide whether and how to integrate postpartum family planning into rural infant vaccinations camps offered by the public health system in India. Interviews explored stakeholder perceptions regarding factors that can influence successful implementation of linked family planning and infant vaccination care in rural India. From March to May 2022, we collected one-time semi-structured interview data from participants (N = 60) recruited from rural subdistrict Junnar in the Pune district of Maharashtra, India. There are 12 primary health centers (PHCs) in Junnar, each serve 10–20 villages. Each village has a community center where monthly vaccination camps are held, usually during village health and nutrition days (VHNDs).

Our sample included postpartum married women (n = 20); husbands (n = 10) and mothers-in-law (n = 10) of postpartum women; frontline healthcare workers from the public health system (auxiliary nurse midwives (ANMs), Accredited Social Health Activists (ASHA)) (n = 10); and community leaders (village panchayat leaders, physician medical officers, and managers in non-governmental organizations engaged in delivery of family planning in the region) (n = 10). Physician medical officers can be both frontline healthcare workers and community leaders in this setting depending on their clinical and leadership role. Our research field staff was comprised of Masters-level social workers and psychologists trained in study protocols as well as qualitative data collection and analysis. Staff were gender-matched to those they were recruiting, and the same team member then collected the data from participants they recruited into the study. The research team member approached eligible postpartum women, husbands, and mothers-in-law near antenatal clinics, primary health centers, community centers, and vaccine camps and offered participation in the study. Members of the same family (women, husbands, and mothers-in-law) were eligible to participate but we did not intentionally recruit members of the same family or link data in any way between family members. Women greater than 12 weeks postpartum and husbands of women greater than 12 weeks postpartum were excluded. Frontline healthcare workers and community leaders were approached and offered participation at their place of work or community centers. We also used snowball sampling and other referral systems to reach the desired number and types of participants.

Once an individual was identified as eligible and willing to participate in the study, the research staff members invited the individual to a private location scheduled near time and location of recruitment, at a time convenient to the participant, to participate in an interview that lasts approximately 40-minutes. Participants provided written informed consent immediately prior to the interview, which we audiotaped for transcription and data analysis.

Across participants, our interview guides focused on feasibility and acceptability of the proposed integration of family planning counseling into the vaccination camps, and the feasibility and acceptability of provision of all reversible contraceptive methods in the community setting. We included probes exploring provision of intrauterine devices (IUDs) in particular given the additional training and privacy required to provide this highly effective reversible method. We defined feasibility as the extent to which an intervention can be carried out in this specific setting, and acceptability as the perception that an intervention will be accepted by the population or community [21]. Among postpartum women, we also assessed experiences with contraceptive counseling, consent, and receipt of contraceptives. Among frontline health workers and community leaders, we additionally explored perceived barriers to incorporating family planning provision into the existing community health infrastructure, and recommendations for intervention development. Probes further explored how community-based care might affect interpersonal quality of care. To better understand the data on potential intervention implementation, we utilized the Consolidated Framework for Implementation Science Research (CFIR) to structure the interview guide and analysis [22], focusing on the five key domains:

1. *inner setting* (i.e., the culture, available resources, and implementation climate),
2. *outer setting* (i.e., political and organizational context where the intervention occurs),
3. *intervention characteristics* (i.e., features of the intervention that affect implementation such as cost, strength of the evidence for the intervention, adaptability),
4. *participant characteristics* (i.e., attitudes and preferences of the intervention participants), and
5. the *process of implementation* (i.e., planning, leadership, and execution of the intervention).

After completion of the interviews, research staff provided community members with information regarding where to receive family planning services in the local public health system. Digital audio was saved electronically with only an ID; no identifiable information was retained for any participants.

We sequentially analyzed data throughout data collection to refine probes and assess for thematic saturation. Research interviewers translated and transcribed audiotaped interviews and uploaded these transcriptions to ATLAS.ti for analysis (version 22). The investigator team reviewed a subsample of 10 interviews with research staff to generate a codebook for data analysis, inclusive of the CFIR domains and identified themes within these domains; the codebook was refined through iterative analysis and discussion. Two coders then independently analyzed the first 10 interviews, and inter-coder reliability was 89%. Only one team member coded each transcript subsequent to these first 10 interviews, though the lead investigators spot-checked coding as part of quality control efforts.

The University of California, San Diego and Sigma India institutional review boards approved this study protocol.

## Results

Postpartum women were 20–31 years old. Participating husbands were 27–42 years old. Frontline health workers interviewed included five ANMs and five ASHAs. Local community leaders included four village panchayat leaders and two NGO workers (three men and three women). There were four medical officers (two men and two women) who were both community leaders and frontline healthcare workers. Participants were included from villages across all 12 primary health center regions.

Three primary themes emerged: (1) Social fertility and gender norms such as son preference and male control over contraceptive decision-making influence postpartum contraceptive access and choice. (2) Linking contraceptive care and infant vaccination is perceived as potentially feasible and acceptable to implement to families, health workers, and community members. (3) Barriers and facilitators to linked infant postpartum contraception and infant vaccination were identified across the five CFIR domains. These themes are described in detail below.

### Social fertility and gender norms influence postpartum contraceptive access and choice

Gender norms are widely held beliefs about gender roles, standards or expectations that influence behavior. We found that gender-inequitable norms influence contraceptive use and agency to use postpartum contraception. Our data suggest that traditionally women in the community are expected to demonstrate fertility early in marriage and not use contraception until the number and desired sex of children is achieved. We also found that traditionally men control the final decision making on fertility and contraception decisions. We found that very few couples in this region of rural India use reversible contraception to achieve healthy birth spacing. Instead, permanent female contraception is the most used method of contraception, and it is typically sought once the desired number and sex composition of children are achieved. One mother-in-law reported, “What contraceptives should be used to keep the spacing between the second and the first child? This is not discussed. So, after three or four children, sterilization is done immediately” [mother-in-law #2, age 50]. However, some participants expressed a historical change towards greater control by women over informed fertility and decision making, “In our time there was no such thing available [contraception] …. now a days women know when to get pregnant and how not to” [mother-in-law #2, age 50].

We found that son preference norm often influences fertility goals. “They tell us before delivery if they have a male baby, then they prefer to have sterilization” an ASHA explained [ASHA#4, age 44], and “the husband might pressure her to have a male child” a husband [husband #2, age 31] shared. When discussing family composition, a mother-in-law said, “I think there should be a boy” [mother-in-law #2, age 50] in the family.

We found that family members, particularly mothers-in-law, influenced couples’ contraceptive decision making. “There is pressure from mothers-in-law not to use contraceptive method” one ANM noted [ANM #2, age 44]. “She may be under pressure from her family to have a baby” [husband #5, age 33] and “there can be pressure from the in-laws” [husband #6, age 35] participants described. One participant said, “She cannot act on her own. She has pressure from home. If a woman wants to make a decision at home, she has to get everyone’s permission. She is under pressure from her in-laws” [NGO worker #2, age 28].

Many participants felt that men should be engaged in family planning decision making. Some women reported needing permission /approval from their husbands for postpartum family planning use. Participants noted, “men should take an active role in family planning,” [husband #10, age 36] and “as head of the family I make all the decisions” [husband #9, age 27]. One mother-in-law explained about choosing a contraceptive method, “she will have to consult her husband” [mother-in-law #4, age 60].

### Linking contraceptive care and infant vaccination is perceived as potentially feasible and acceptable to implement by families, health workers, and community members

Participants were asked about the theoretical feasibility of providing postpartum contraception at the time of infant vaccination in their community. Many participants confirmed that “women are the ones who bring the babies for vaccination” [ANM #2, age 44]. They discussed that often husbands and other family members accompany women. ASHAs often provide care to women in their homes during and after pregnancy and facilitate their attendance at community vaccination camps. Multiple ASHAs and ANMs shared concerns about privacy when doing contraceptive counseling during home visits and reported that the vaccination camps could be a more ideal place to provide contraceptive care. “There is not privacy for every woman at home because other family members are present in the house which makes them reluctant to ask their doubts. Here [at the community center / vaccination camp], she can tell you” [ANM #1, age 28].

Healthcare workers and families were optimistic that the camp provided a time and place where private contraceptive counseling could be done, and contraception could be provided. “We can provide what women need through the camp”, an ASHA described, “for example, if anyone wants a cuT [Copper IUD] we can provide it to her” [ASHA #1, age 31]. The ANMs described a system where infants and their families are often called “one-by-one” for the vaccination [ANM #2, age 44]. Most health care providers confirmed that women were present at the time of infant vaccination and that husbands also often attend the camp but are not always in the vaccine visit. “Husbands also come,” one postpartum woman described, “they wait outside sometimes come inside if are called” [woman #3, age 21]. “Women are the only ones who bring babies for vaccination, so you have [privacy] to tell them at that time” [ANM #2, age 44] an ANM reported. However, a husband shared that they do sometimes accompany women to the vaccination camp, “when it comes to vaccination or weight check, their husband comes with them” [husband #9, age 27] he said.

Participants were also asked about the theoretical acceptability of providing postpartum contraception at the time of infant vaccination in their community. Many participants expressed that providing family planning at the time of infant vaccination would be acceptable—providing care to women and families where they are in their community. Most participants agreed that the intervention would be beneficial to community. “It will definitely benefit the beneficiaries because they frequently visit center for vaccination and less likely to visit in future for any other reason” an ANM [ANM #1, age 28] shared. “Because all these people have come, there will be a discussion. Their baby is small at the time of vaccination, and they are also looking for family planning services. They may be looking for information and it will be much easier and more convenient if information and methods are available at the time of vaccination” a husband [husband #8, age 28] described. “Counselling on family planning is needed. Such family planning services have not yet reached in such villages” another participant [woman #10, age 36] reported.

### Barriers and facilitators to linked infant vaccination and postpartum contraception were identified across the CFIR domains

#### Inner setting (i.e., the culture, available resources, and implementation climate)

Resource barriers identified in the inner setting domains include limited time, staff, and space, particularly where nursing staff vacancies are common. Limited numbers of nursing staff in the setting of nursing shortages was identified as the greatest barrier to providing adequate time to counsel women and couples in private and provide contraception if desired. Many ANMs and ASHAs felt that this model of linked care could be facilitated by ensuring availability of additional staff and a private room. One ANM shared, “in our culture, people are not free to discuss private matters…if we want to provide these services, we do not have extra staff to give them all this information. When we want to give this information personally on family planning methods, we require separate room to maintain confidentiality to resolve all her doubts. In short, there should be a separate staff and room for all this so that there should be privacy” [ANM #1, age 28]. She continued, “there will need to be an independent health worker to give this information at the time of vaccination and then we can take the couple to a separate room and give this information” [ANM #1, age 28].

#### Outer setting (i.e., political and organizational context where the intervention occurs)

The need for health centers and providers to meet family planning provision goal targets was seen as a barrier to expanding access to the full range of contraceptive options in a patient-centered way. Women are incentivized to accept sterilization and ASHAs are incentivized if they counsel women who accept sterilizations. However, these incentives were unavailable for most other methods of contraception. “Those who have undergone such operations, they get 300 rupees and we also get 150 rupees” an ASHA [ASHA #1, age 31] explained.

The cultural focus on family planning to support healthy birth spacing was seen as a facilitator to a new program expanding access to family planning. “If family planning services are provided,” shared a medical officer, “the population growth can be stopped, and their children can be well cared for. That is, the next generation is healthy and capable” [medical officer #1, age 36].

#### Intervention characteristics (i.e., features of the intervention that affect implementation such as cost, strength of the evidence for the intervention, adaptability)

Provision of contraception at the infant vaccination camps, allowing co-location of maternal and infant services was seen as a facilitator of successful intervention delivery. This intervention leverages the frequent infant vaccine dosing schedule allowing for multiple opportunities for contact to support women and couples in shared decision-making around contraceptive care reaching them in their communities without requiring travel. “Now look at my village, it is a farming community, people don’t go to the hospital,” a medical officer shared, “we provide those services [family planning] but they can’t take advantage of it” [medical officer #4, age 32].

Participants felt delivery of contraception in this setting allowed for possible male engagement in family planning decision-making. “If both [husband and wife] are face-to-face through the camp it will be more convenient to give information to both. If both are together, they can find answers to every question in the camp. Family planning methods are available, no need to go anywhere, camps can be convenient for everyone” [husband #2, age 31] a participant explained.

#### Characteristics of individuals (i.e., attitudes and preferences of the intervention participants)

Inequitable gender norms in contraceptive decision making were perceived as an important determinant of implementation success. Many participants shared that women in the community do not typically make contraceptive decisions without their husbands and were less likely to use contraception without the presence and approval of their husbands. However, since husbands frequently attend infant vaccination visits, their presence could support women to initiate contraception at that time if desired. “There is hardly a woman who does not tell her husband but there are many who will not do anything without telling her husband,” [ANM #3, age 57] a participant shared. An ANM explained, “if she has talked to her husband or other family members about this before, we can insert her CuT there right away, give her all the information she needs. But she might say that she needs to take permission from her mother-in-law or her husband. If they have any problems, they have to go home and talk to the family members. There may be some family problems or if the woman is not able to make a decision on her own, then she does not take it” [ANM #1, age 28]. Women sometimes chose to use contraception covertly or without involving her husband in the decision. ANMs will need to navigate with women whether they want to engage their husbands in contraceptive decision making or whether they want to discuss while their husband is outside or not present.

Sometimes autonomy in reproductive decision making was viewed as a benefit afforded to educated women only and family support for reproductive decision-making was often decisive. “A woman can make decisions if she is educated, but she needs the support of her family. Suppose a woman in the household is uneducated, she cannot decide for herself. Education is very important in this” a participant reported [woman #16, age 25].

#### Process of implementation (i.e., planning, leadership, and execution of the intervention)

Multiple participants agreed that not having support of the community leaders would be a barrier to implementation of this intervention. One village leader explained that the program should be, “implemented jointly with the Gram Panchayat [village council], Government officials and the Health Department.” [village leader #1, age 57]. Participants recommended engaging community leaders to champion the intervention and thereby increase uptake and acceptability. An NGO worker agreed, “if you are going to a particular village, Gram Panchayat members should be informed in advance and then carry out the activities with their help” [NGO #1, age 30]. Many women and husbands recommended the use of media in order to inform the community about the intervention and family planning methods available in advance of the vaccine camp—promoting the program through the ASHAs during prenatal care, through posters in community centers, and even television, newspapers, and social media to generate both awareness and acceptance of the intervention.

## Discussion

We found that linking provision of postpartum contraception to infant vaccination care is feasible and acceptable in rural India, conditional on addressing resource-related barriers to family planning provision in this setting.

Similar to other studies, we found that gender-inequitable norms including son preference and limited mobility of young women to reach health clinics are barriers to accessing and using postpartum family planning in rural India [23, 24], limiting opportunities for male involvement in contraceptive decision-making [25], and facilitating male control over reproductive decision-making [26].

Our findings suggest that an intervention aimed at meeting the reproductive health needs of women in India should also consider gender-equity informed family planning counseling engaging women and communities on issues related to reproductive autonomy and the value of women and girls in society. It is important that this counseling is accessible to couples with a wide range of educational attainment. Our findings suggest that successful family planning interventions in rural India should address community social norms, be inclusive of husbands and mothers-in-laws, and also engage men in the process of family planning decision making. Clinical interventions should happen in parallel to interventions aimed at affecting social norms change. [Theme 1: Social and gender norms influence postpartum contraceptive access and choice].

Co-locating the services in the vaccination camp setting potentially offers an opportunity to reach postpartum women outside of their homes or health clinics where they are in their communities. The infant vaccine dosing schedule, including three vaccine visits that occur over an eight-week period, offers multiple opportunities for outreach, recruitment and follow-up for women and couples in the first months after delivery.

Family planning decision-making is a process. Women and couples often need multiple counseling opportunities to get information about and choose a method that aligns with their preferences and meets their goals in the postpartum period [27]. Providing postpartum contraception at the infant vaccine visit leverages the frequent infant vaccine dosing schedule, allowing for multiple opportunities for contact to support women and couples in shared decision-making around family planning after birth. Offering the option of multiple opportunities to engage with family planning providers could remove perceived barriers to contraception use in the postpartum period. Prior research demonstrated that screening for interest in family planning services at village health and nutrition days where vaccinations occur in India was associated with an increase in women later seeking family planning services through the public health system [15]. We propose a strategy to utilize the infant vaccination visit as an opportunity to reach postpartum women in the community to provide family planning care, including all reversible contraceptive methods through integrated maternal-infant health care visits [Theme 2: Linking contraceptive care and infant vaccination is perceived as potentially feasible and acceptable to implement by families, health workers, and community members].

Engaging participants in intervention design, including different levels of healthcare personnel and community members, allowed us to identify multilevel barriers and facilitators to linked infant vaccination and postpartum family planning. For example, we identified the importance of maintaining privacy for contraceptive counseling and care. We identified that private space for family planning counseling and care must be set aside from the vaccination area to facilitate delivery of respectful high-quality family planning care. We identified that nurses who administer the vaccines are busy and there are frequent vacancies in their posts. Therefore, additional personnel would be needed at the vaccination camps to appropriately implement this intervention in this setting. Given the nursing shortage globally and in India, these findings align with plans to increase the nursing workforce in India [28].

In addition, our data suggests that engaging community leaders, such as the gram panchayat, will be vital to successful community integration. We identified that creating awareness about the program and family planning methods available for postpartum contraception, such as through the ASHAs or social media, is an important component of an implementation strategy that meets the needs of the community. Finally, our data suggest that family planning programs should consider using outcome measures that focus on quality of care in addition to contraceptive uptake rates to measure intervention success and should move away from contraceptive target goals for providers and health centers. [Theme 3: Barriers and facilitators to linked infant vaccination and postpartum contraception were identified across the CFIR domains].

Our study has several strengths. First, we utilized an implementation science framework, CFIR, to guide our data collection and analysis, ensuring that our data can directly inform intervention design and delivery. Second, we engaged a broad range of participants including ASHA healthcare workers, ANMs, postpartum women, husbands and mothers-in-laws of postpartum women, and community leaders and advocates to ensure that the data was informed by multiple perspectives across the community.

Our data should be considered in the context of several limitations. Like most qualitative data, the findings are generalizable only to the community of interest in this region of rural India. Because of the investigators’ commitment to the study goals and some participants’ proximity to, and connection with, vaccination and family planning clinics, our study sample may have included participants who were more knowledgeable and/or supportive of family planning care that the typical community member. Our data relies on self-report and therefore may be subject to social desirability bias. This data was collected during the COVID-19 pandemic and public health response which may have influenced participants perceptions of the healthcare system and the feasibility of this intervention. Most importantly, our data explores the theoretical feasibility and acceptability of linking infant vaccination and family planning care. Further pilot data is needed to understand the actual effect of integrating these infant and maternal services on community and individual health outcomes.

## Conclusions

Linked provision of family planning and infant vaccination care may be feasible and accessible in rural India utilizing strategies identified to reduce barriers and facilitate provision of care. The benefits of linking family planning to infant vaccination are that the intervention provides opportunities for women and couples to receive care in their communities with multiple opportunities for discussion and follow-up. Facilitators to linking family planning and infant vaccination include engaging men and community leaders in the program. Potential barriers include limited time and space resources in the public health system. Elements of a successful implementation strategy identified were to include gender-equity focused counseling and securing dedicated personnel for counseling prior to implementing the program. These findings have the potential to guide implementation of an innovative patient-centered gender-transformative family planning intervention which increases access to contraception for postpartum women with unmet need.

## Data Availability

The de-identified data analyzed are available from the corresponding author on reasonable request.

## List of Abbreviations

ASHA: Accredited Social Health Activist
ANM: Auxiliary nurse midwife
CFIR: Consolidated Framework for Implementation Research
CuT: Copper “T” intrauterine device
IUD: Intrauterine device
VHND: Village health and nutrition day
